# Association of endothelial nitric oxide synthase promoter region (T-786C) gene polymorphism with acute coronary syndrome and coronary heart disease

**DOI:** 10.1186/1476-511X-7-5

**Published:** 2008-02-25

**Authors:** Ç Çiftçi, S Melil, Y Çebi, M Ersöz, P Çağatay, M Kılıçgedik, B Süsleyici Duman

**Affiliations:** 1 Istanbul Science University, Faculty of Medicine, Department of Cardiology, Istanbul, Turkey; 2 Istanbul Science University, Faculty of Medicine, Department of Medical Biology and Genetics, Istanbul, Turkey; 3 Istanbul Science University, Faculty of Medicine, Basic Sciences Laboratory, Istanbul, Turkey; 4 Istanbul University, Cerrahpasa Faculty of Medicine, Department of Biostatistics, Istanbul, Turkey

## Abstract

**Background:**

Nitric oxide (NO) is an endothelium derived relaxing factor (EDRF) which has an important role for regulating the heart-vessel physiology. The objective of this study was to evaluate the effects of the eNOS T-786C polymorphism on lipid parameters and the development of acute coronary syndrome (ACS) and coronary heart disease (CHD) for the first time in a Turkish study group. We have analyzed the genotype frequencies of the T-786C polymorphism of the eNOS gene in 10 ACS patients (5 men, 5 women), 20 CHD patients (14 men, 6 women), and 31 controls (10 men, 21 women), who were angiographically proven to have normal coronaries.

**Results:**

The demographic, biochemical and left ventricule systolic dysfunction data of the ACS, CHD patients and controls were analyzed as a function of eNOS T-786C genotypes. The eNOS gene T-786C polymorphism frequencies for T/T, C/T and C/C genotypes were respectively 10%, 40%, 50% in subjects with ACS; 75%, 20%, 5% in subjects with CHD and 67.7%, 25.8%, 6.5% in the control group. Significant difference was observed in genotype frequencies between the study groups for T-786C polymorphism (p = 0.001). The CC genotype frequency was found to be the most prevalent in ACS group in comparison to CHD and control groups (p = 0.001). TT was the most frequently observed genotype in both CHD patients and controls (p = 0.001). Left ventricule systolic dysfunction frequency was found to be highest in C/T genotype carriers (66.7%) in patients (ACS+CHD). None of the patients with LVSD were carrying the normal genotype (T/T). The eNOS T-786C polymorphism was not found to be effective over any analyzed lipid variable in patients (ACS+CHD). The HDL-cholesterol levels were found to be lower in CHD group were compared to controls (p < 0.01), whereas glucose and leucocyte levels of the ACS and CHD groups were both higher than controls (p < 0.001).

**Conclusion:**

The significantly high frequency of eNOS -786C/C genotype in ACS patients than in those of controls, indicate the genotype association with ACS. The finding of significantly high frequency of T/T genotype in the CHD group, may support the relationship of CC genotype with ACS without CHD. The high frequency of the mutant (C/C) and heterozygous (C/T) genotypes found may be linked to left ventricule remodeling after MI.

## Background

Nitric oxide (NO) is a key factor in the antiatherosclerotic properties of the endothelium. NO regulates vascular smooth muscle cell proliferation and migration, vascular tone, endothelial permeability, and endothelial-leucocyte interaction, and has antithrombotic effects [1-4]. Genetic polymorphisms of eNOS have been shown to have a significant effect on NO levels, plasma lipids and have been associated with T2DM [5], heart failure [6], coronary spasm [7], atherosclerosis [8], myocardial infarction [9], coronary in-stent restenosis [10] and hypertension [9] in some studies. Because of the pleiotrophic effects of NO, various studies have investigated the link between polymorphisms of the endothelial nitric oxide synthase (eNOS) gene and the development of coronary events. Among the many reported polymorphisms of the eNOS gene, two polymorphisms, namely the Glu298Asp(G:T) polymorphism located in exon 7, and T-786C in promoter have received much interest with respect to the possible association between such polymorphisms and coronary artery disease (CAD) [11,12]. In the present study, we sought to evaluate the effects of the eNOS T-786C polymorphism on the development of ACS and CHD.

## Materials and methods

### Study subjects

We studied 10 ACS patients (5 men, 5 women), 20 CHD patients (14 men, 6 women), and 31 controls (10 men, 21 women), who were angiographically proven to have normal coronaries from Group Florence Nightingale Hospital (Istanbul, Turkey). Patients who had unstable angina pectoris (USAP), ST-elevation myocardial infarction (STEMI) and non-STEMI (NSTEMI) were referred as ACS. All the ACS patients had chest pain longer than 20 min, in resting state. All ACS patients serum cardiac enzymes (troponin T, creatine kinase-MB subforms) were high. All ACS patients has ischaemic ST-T changes on electrocardiography. The criteria for CHD was narrowing of artery 50% or more with angiography, who had stable angina pectoris and who were ischaemia positive with noninvasive tests. Left ventricule systolic functions were evaluated with echocardiography. Patients with ejection fraction (EF) between 35 and 50 were accepted to have moderate left ventricule systolic dysfunction (LVSD), whereas below 35 was accepted as severe LVSD. Since only 2 patients had severe LVSD, only 1 patient with moderate LVSD were included in the statistical analysis. The mean EF of the patiens (ACS+ CHD) was 37.4 ± 1.85. Conventional risk factors for CHD such as dyslipidemia (HDL-cholesterol levels <45 mg/dl, triglyceride levels >150 mg/dl and LDL-cholesterol levels>130 mg/dl), hypertension (blood pressure > 130/80 or prior therapy), diabetes mellitus (fasting blood glucose of >120 mg/dl or prior therapy), obesity (BMI >25), positive family history for CHD and smoking (current smokers) were obtained by viewing records and interviewing patients. The mean age was 60.20 ± 2.52 for ACS patients, 60.70 ± 1.99 for CHD patients and 59.00 ± 1.68 for controls. Written consent was taken from each patient following a full explanation of the study, which has been approved by the Ethics Committee of the Istanbul Science University. The study groups were matched for age, as well as social and economic status.

Subjects with secondary hypertension (renal artery stenosis, glomerulonephritis), diabetic nephropathy (Kimmelstiel-Wilson syndrome), hypertension with endocrinopathies (phoechromocytoma, Cushing syndrome, hyper and hypothyroidism), patients with pseudohypertension, neoplasia and those who take oral contraceptives and illicit drugs were not included in the study.

### Analytical methods

The plasma glucose concentration was measured by the glucose oxidase method using Kit of Biotrol on Bayer/opeRA analyser. Serum Total-Cholesterol was measured using commercial kit of Biotrol; HDL-Cholesterol using by commercial Randox's kit; LDL-Cholesterol was calculated by the formula of Friedewald) and triglyceride determination was made by the method of lipase/glycerol kinase UV endpoint on opeRA analyser.

### DNA Extraction and Genotyping

Blood was drawn from the antecubital vein into EDTA. Genomic DNA was extracted from leukocytes by a salting out procedure [13]. Polymerase chain reaction (PCR) and restriction fragment length polymorphism analysis was used for genotyping T-786C polymorphism [14]. Genotyping was conducted in a blinded fashion. A total of 10% of samples were subject to repeat PCR and genotyping, and no discrepancies were detected.

### Statistical analysis

Statistical analyses were conducted using the Unistat 5.1 software program. Data were expressed as means ± SE. Baseline differences between patients and controls were examined by Student t-test. Hardy-Weinberg equilibrium for genotype frequencies was estimated by the Chi-square test. The Bonferroni correction for multiple testing was applied as required. P values less than 0.05 were considered significant.

## Results

The clinical characteristics of ACS patients, CHD patients and control subjects were compared in Table 1. Groups were statistically different when compared with chi-square test with respect to hypertension (p = 0.0001), diabetes mellitus (p = 0.0001), dyslipidemia (p = 0.009), heredity (p = 0.0001), recent myocardial infarction (MI) (p = 0.002), left ventricule systolic dysfunction (p = 0.0001), left ventricule diastolic dysfunction (p = 0.016) and left ventricule hypertrophy (p = 0.004) (Table 1).

**Table 1:** Clinical characteristics of acute coronary syndrome and coronary heart disease patients and control subjects

	**ACS n(%)**	**CHD n(%)**	**Controls n(%)**	**p**
	
**Hypertension**	9 (90.0)	15 (75.0)	2 (6.5)	0.0001
**Diabetes mellitus**	4 (40.0)	10 (50.0)	0 (0)	0.0001
**Dyslipidemia**	9 (90.0)	10 (50.0)	10 (34.5)	0.009
**Obesity**	1 (20.0)	8 (57.1)	15 (50.0)	0.40
**Smokers**	4 (40.0)	12 (60.0)	9 (29.0)	0.10
**Heredity**	7 (70.0)	10 (50.0)	1 (3.2)	0.0001
**Recent myocardial infarction**	4 (40.0)	2 (10.0)	0 (0)	0.002
**Left ventricule systolic dysfunction**	4 (40.0)	2 (10.0)	0 (0)	0.0001
**Left ventricule diastolic dysfunction**	9 (90.0)	16 (80.0)	0 (0)	0.016
**Left ventricule hypertrophy**	3 (30.0)	4 (20)	0 (0)	0.004

The variables were compared with χ^2 ^test among groups.

The genotype frequency distributions of ACS, CHD patients and 31 control subjects with respect to T-786C polymorphism was compared in Table 2. The eNOS gene T-786C polymorphism frequencies for T/T, C/T and C/C genotypes were respectively 10%, 40%, 50% in subjects with ACS; 75%, 20%, 5% in subjects with CHD and 67.7%, 25.8%, 6.5% in the control group. Significant difference was observed in genotype frequencies between the study groups for T-786C polymorphism (p = 0.001). In detail, when the ACS, CHD and control groups were compared with respect to eNOS T-786C genotypes, the CC genotype frequency was found to be the most prevalent in ACS group in comparison to CHD and control groups (p = 0.001). Whereas TT was the most frequently observed genotype in both CHD patients controls (p = 0.001).

**Table 2:** Endothelial nitric oxide synthase gene T-786C genotype frequencies in acute coronary syndrome and coronary heart disease patients and control subjects

	**eNOS gene T-786C genotypes**		
	**T/T;n(%)**	**C/T;n(%)**	**C/C;n(%)**
	
**Acute coronary syndrome**	1(10)	4(40)	5(50)*
**Coronary heart disease**	15(75)*	4(20)	1(5)
**Control**	21(67.7)*	8(25.8)	2(6.5)

Genotype frequencies were compared with χ^2 ^test. * p = 0.001.

Distribution of left ventricule systolic dysfunction (LVSD) in ACS and CHD patients as a function of eNOS gene T-786C genotypes were presented in Table 3. Left ventricule systolic dysfunction frequency was found to be highest in C/T genotype carriers (66.7%) in patients (ACS+CHD). None of the patients with LVSD were carrying the normal genotype (T/T) (Table 3).

**Table 3:** Distribution of left ventricule systolic dysfunction in acute coronary syndrome and coronary heart disease patients as a function of eNOS gene T-786C genotypes

	**eNOS gene T-786C genotypes**		
	**T/T;n(%)**	**C/T;n(%)**	**C/C;n(%)**
	
**LVSD**	0 (0)	4 (66.7)	2 (33.3)

LVSD: Left ventricule systolic dysfunction. Genotype frequencies were compared with χ^2 ^test. p = 0.011. 2 patients with severe LVSD (Ejection fraction <35) were not included in the analysis. Only 1 patient with moderate LVSD (Ejection fraction between 35–50) were included.

The demographic and biochemical data of the ACS patients, CHD patients and control subjects were given in Table 4. The HDL-cholesterol levels were found to be lower in CHD group were compared to controls (p < 0.01), whereas glucose and leucocyte levels of the ACS and CHD groups were both higher than controls (p < 0.001) (Table 4).

**Table 4:** Demographic and biochemical data of the acute coronary syndrome and coronary heart disease patients and control subjects

	**ACS**	**CHD**	**Control**
	
**BMI (kg/m^2^)**	26.50 ± 1.69	28.97 ± 1.26	26.04 ± 0.75
**Glucose (mg/dl)**	134.13 ± 26.21 ^b^	126.80 ± 9.95^b^	66.97 ± 2.26
**Leucocyte (count/mm^3^)**	10466.67 ± 1075.48 ^b^	8825.88 ± 426.73 ^b^	4946.77 ± 154.92
**Total-cholesterol (mg/dl)**	216.10 ± 15.84	207.53 ± 10.42	198.69 ± 8.70
**HDL-cholesterol (mg/dl)**	43.70 ± 4,50	39.00 ± 2.29^a^	47.11 ± 1.87
**LDL-cholesterol (mg/dl)**	136.90 ± 12.54	134.79 ± 9.16	125.89 ± 7.48
**Triglyceride (mg/dl)**	152.50 ± 18.08	168.74 ± 17.87	137.14 ± 9.27

Values are represented as mean ± SD. a: p < 0.01 in comparison to control group, b: p < 0.001 in comparison to control group

The lipid parameters of the patients (ACS+CHD) are compared in Table 5 as a function of eNOS T-786C genotypes. The eNOS T-786C polymorphism was not found to be effective over any analyzed lipid variable (Table 5).

**Table 5:** Associations of lipid parameters with endothelial nitric oxide synthase gene eNOS T-786C genotypes in the patiens (acute coronary syndrome and coronary heart disease patients)

	**eNOS T-786C genotypes**		
	**T/T (n = 16)**	**C/T (n = 8)**	**C/C (n = 6)**
	
**Total-cholesterol (mg/dl)**	207.00 ± 39.81	216.75 ± 42.38	210.83 ± 70.63
**HDL-cholesterol (mg/dl)**	41.07 ± 13.60	38.50 ± 9.46	42.33 ± 12.69
**LDL-cholesterol (mg/dl)**	138.07 ± 41.48	134.63 ± 24.52	130.33 ± 53.46
**Triglyceride (mg/dl)**	164.13 ± 83.70	151.88 ± 53.54	165.67 ± 64.53

## Discussion

Investigations into the relation between eNOS gene polymorphism with ACS and CAD have given various and sometimes contradictory results. Fatini et al. [15] provided evidence that the -786CC pattern modulates the susceptibility to ACS in 4a4a homozygotes and in hyperhomocysteinemic patients. In the Ukrainian population Dosenko et al. [16] showed that the CC genotype of the T-786C polymorphism were found 2.7 times more often in ACS patients than in controls, and thus considered its allelic polymorphism as one of genetic risk factors of ACS development. The findings of Nakayama et al.[17] have strongly suggested that the CC variant in the T-786C polymorphism of eNOS gene reduced the eNO synthesis and predisposes the patients with the mutation to coronary spasm. Additionally the T-786C polymorphism in combination with smoking have been reported to increase the risk of coronary spasm in several studies in Japanese patients [18,19]. In another study of Nakayama et al. [20], the frequency of the T-786C mutation was found to be significantly higher in MI patients with no stenosed vessels (50%) than in those with stenosed vessels (p < 0.003), and concluded the T-786C mutation in strong association with MI, especially without coronary arterial stenosis, in Japanese patients. In the present study, the frequency of eNOS -786C/C genotype was found to be significantly higher in ACS patients (50%) than in those of controls (6.5%), which indicate the association of -786C/C genotype with ACS. Our finding of significantly high frequency of T/T genotype in the CHD group, may support the relationship of CC genotype with ACS without CHD.

Alvarez et al. [21] found eNOS-CC+ACE-DD at a higher risk for early CAD, whereas the GENICA study performed on Caucasions have found the C allele to be associated with increased risk of multivessel CAD [22]. A follow up GENICA cohort study [23] performed on high risk CAD patients evaluating cardiovascular mortality found that, the T-786C SNP in the promoter of eNOS beared independent prognostic information with oxidant stres markers. A meta analysis performed over 26 studies involving 23028 subjects reported lack of influence of T-786C variant on ischaemic heart disease (IHD) risk, but a very small effect of the variant cannot be excluded, since they found only a 73% power to detect an OR of 1.2 at a significance level of 5% [24]. Gomma et al. [10] have reported T-786C polymorphism to be associated with coronary in-stent restenosis in patients with CAD. In detail, they found that carriers of the -786C allele of the eNOS T-786C polymorphism showed a higher frequency of restenosis [10]. In another study, C allele homozygosity in position -786 of the eNOS promoter has been detected to be an independent risk factor for moderate to severe internal carotid artery stenosis, especially ulcerative lesions [25]. In our study, the mutant genotype (CC) frequency of T-786C polymorphism was found in low percentage (5%) to that of wild type (TT) (75%) and heterozygous (CT) (20%), which indicates that no association persists between T-786C variation and CHD whereas, the frequency distributions of eNOS T-786C genotypes were similar in CHD and control groups in the present study. In a study performed on Japanese population [19] composed of 209 men and 238 women, the frequencies of coronary spasm respectively in non-smokers with C/T or CC genotype was found to be 61% for male and 78% for females; wheras smokers with C/T or CC genotype was found to be 91% for male and 92% for females, which clearly demonstrated the T-786C polymorphism and smoking in combination increasing the risk of coronary spasm. In our study the eNOS T-786C genotype frequencies were not found to differ significantly with respect to smoking in none of the study groups analyzed (data not included).

In 1106 caucasion multivessel CAD patients Rossi et al. [22] reported T-786C T/T, T/C and C/C genotype frequencies to be %41.9, %40.4 and %17.7. Alvarez et al. [21] reported a significant increase in CC genotype frequency in comparison to C/T and TT in CAD patients. Jeerooburkhan et al. [26] reported significant difference among T-786C genotypes. In detail, the T/T, T/C and C/C genotype frequencies were found to be respectively as %37.7, %47.8 and %14.5 in 3052 middle aged British men initially free of IHD. According to the data of Jeerooburkhan et al. [26] no influence was found between eNOS T-786C polymorphism and the risk of IHD as a result of 8.1 years follow up. Neither Poirier et al. [27] in the French population nor Granath et al. [28] in the Australian Caucasion population did not find significant difference among CAD cases and controls with respect to eNOS T-786C genotype frequencies. Similar to the results of Poirier et al. [27] and Granath et al. [28] we did not observe any significant difference among CAD cases and controls with respect to eNOS T-786C genotype frequencies.

Nitric oxide can modulate many of the processes leading to ventricular remodeling [29]. Endothelium-derived NO causes systemic vascular relaxation [30], thereby reducing cardiac preload and afterload. Recent evidence suggests that NO can increase angiogenesis, decrease cardiac fibrosis, and decrease angiotensin II-induced cardiac myocyte hypertrophy [31], all of which could limit ventricular remodeling after MI. Recently, a eNOS gene polymorphism, G894T, which alters enzyme function [32,33], was associated with an increased risk of CAD [34]. Scherrer-Crosbie et al. [35] have reported the importance of eNOS in limiting LV dilatation, dysfunction, and hypertrophy in murine MI, possibly by limiting the hypertrophic response to MI, and they suggest new strategies for preventing detrimental LV remodeling in patients after MI. In the present study, ACS and CHD patients LVSD with ejection fraction between 35–50%, the eNOS gene -786T/T wild type genotype was not observed, whereas the heterozygous genotype, -786C/T genotype frequency found in highest (66.7%). Since the limitation of this study was the relatively small sample size, the study should be replicated with a larger sample.

In conclusion, The significantly high frequency of eNOS -786C/C genotype in ACS patients than in those of controls, indicate the genotype association with ACS. In addition, the finding of significantly high frequency of T/T genotype in the CHD group, may support the relationship of CC genotype with ACS without CHD. The high frequency of the mutant (C/C) and heterozygous (C/T) genotypes found may be linked to left ventricule remodeling after MI. These findings imply that, although the mechanism underlying the association between the eNOS gene polymorphism and ACS has so far remained elusive, the genetic background controlling nitric oxide may be associated with the pathogenesis of ACS.
